# Supplementary material for: The RIG‐I‐like receptor LGP2 inhibits Dicer‐dependent processing of long double‐stranded RNA and blocks RNA interference in mammalian cells
Source: EMBO J. 2018 Jan 19;37(4):e97479. doi: 10.15252/embj.201797479 (PMC5813259; doi:10.15252/embj.201797479)
Supplement: Supplementary file 1 — Expanded View Figures PDF [file EMBJ-37-e97479-s001.pdf]

# Expanded View Figures

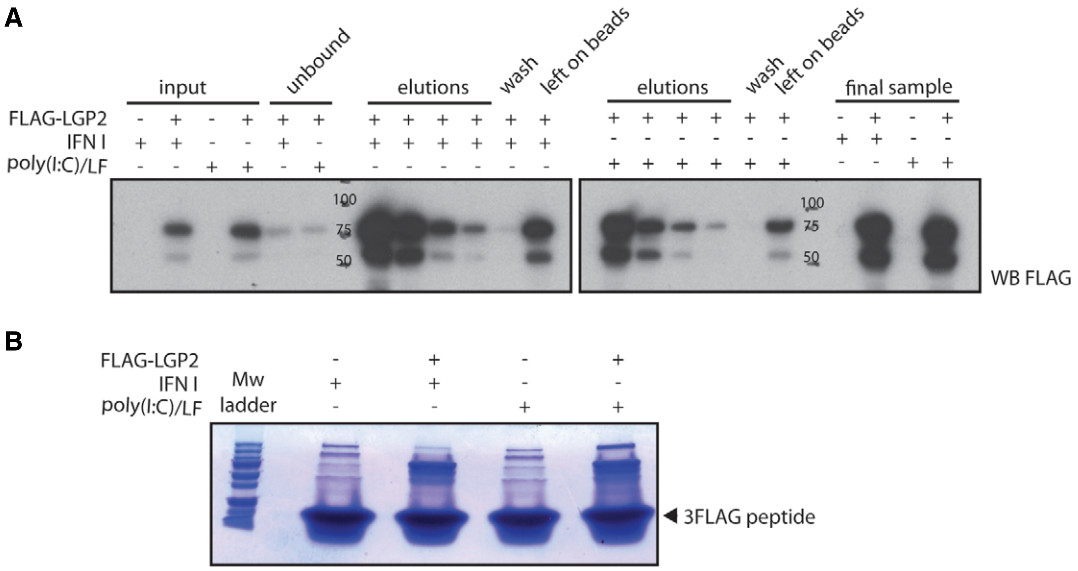


**Figure EV1. Immunopurification of FLAG-LGP2 complexes for LC-MS/MS analysis.**

A FLAG immunoprecipitation followed by peptide elution efficiently retrieved LGP2 complexes from HEK293 cells. Experiment was performed as described in Fig 1A. For input and unbound samples, 0.05% of total lysate was taken prior and post-IP. For all other samples, 1% of the total fraction was loaded. Four elution steps were performed, pooled and concentrated on a Vivaspin 500 column to yield the final sample.

B The concentrated LGP2 complexes were loaded onto a 4–20% polyacrylamide gel, run for a few centimetres into the gel and visualised by Coomassie stain. Eight gel slices per lane were excised for trypsin digestion and further processing for LC-MS/MS analysis.

Source data are available online for this figure.

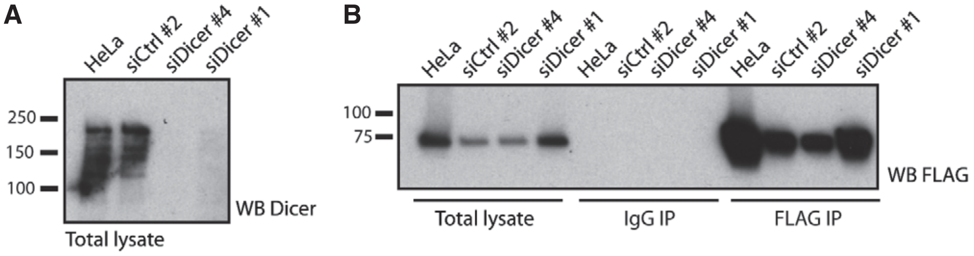


**Figure EV2. siRNA-mediated depletion of Dicer and immunoprecipitation of FLAG-LGP2 from HeLa cells.**

A Dicer is efficiently depleted in HeLa cells following siRNA treatment. Aliquots for immunoblot analysis were taken from the experiment described in Fig 3B.

B LGP2 was efficiently retrieved by FLAG immunoprecipitation from HeLa cells. A fraction of the total lysates and immunoprecipitates from the experiment described in Fig 3B was taken for analysis by anti-FLAG immunoblot.

Source data are available online for this figure.

© 2018 The Authors

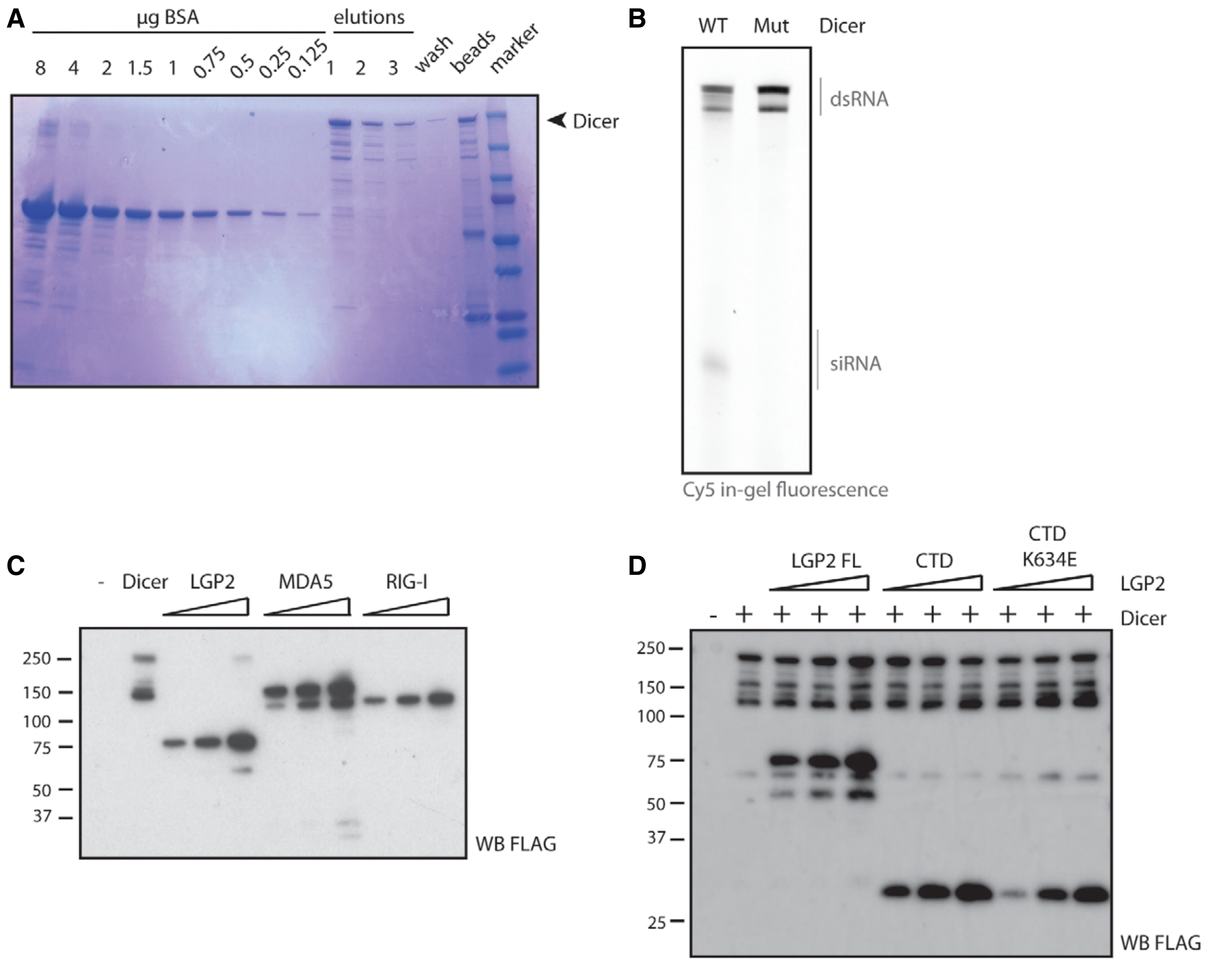


**Figure EV3. Set up of the *in vitro* dicing assays and related controls.**

A Purification of FLAG-tagged human Dicer from HEK293T cells. FLAG-hDicer was expressed in HEK293T cells by transient transfection and subsequently immunoprecipitated using a FLAG antibody, followed by three rounds of elution from the resin using 3FLAG-peptide. Aliquots were analysed by SDS–PAGE and Coomassie staining. The remaining fraction on the beads was tested to verify efficient elution.

B The small RNAs generated in the dicing assay require the catalytic activity of Dicer. Fifty nM dsRNA internally labelled with Cy5 was incubated with 500 nM wild-type FLAG-hDicer or a catalytic mutant (D1320A/D1709A) for 1 h at 37°C prior to analysis on a denaturing polyacrylamide gel by in-gel fluorescence.

C To verify equal protein input into the dicing assay of Fig 4C, an equal amount of protein was taken for analysis by SDS–PAGE and immunoblotting using a FLAG antibody.

D To verify equal protein input into the dicing assay of Fig 4D, a small aliquot of each reaction was analysed by SDS–PAGE and immunoblotting using a FLAG antibody.

 © 2018 The Authors

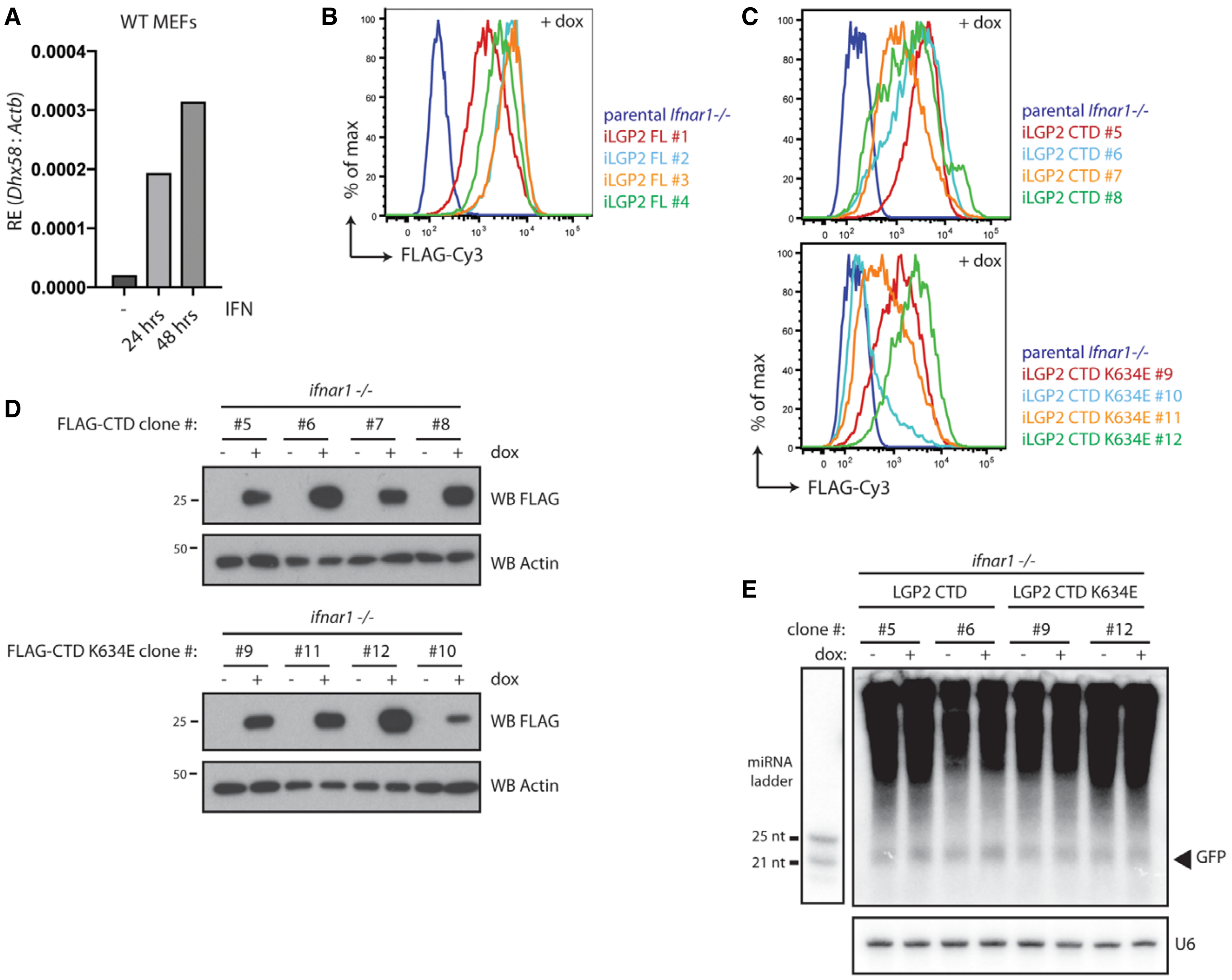


**Figure EV4. Analysis of doxycycline-inducible expression of full-length FLAG-LGP2, FLAG-LGP2 CTD, or FLAG-LGP2 CTD K634E in *Ifnar1*$^{-/-}$ MEFs.**

A Relative expression (RE) of LGP2 (*DHX58*) in wild-type MEFs treated for 24 or 48 h with type I IFN was assessed by qRT–PCR and normalised to β-actin (*ACTB*) using the $\Delta\Delta C_t$ method.

B Verification of doxycycline-dependent induction of FLAG-LGP2 expression in *Ifnar1*$^{-/-}$ MEFs by flow cytometry. Various *Ifnar1*$^{-/-}$ iLGP2 clones were treated for 72 h with doxycycline (dox) and subsequently fixed, permeabilised and stained with a FLAG-Cy3 antibody followed by flow cytometry.

C Verification of doxycycline-dependent induction of FLAG-LGP2 CTD and CTD K634E expression in *Ifnar1*$^{-/-}$ MEFs. The indicated clones were treated for 72 h with dox and subsequently fixed, permeabilised, stained with a FLAG-Cy3 antibody and analysed by flow cytometry.

D Immunoblot analysis of four clones of *Ifnar1*$^{-/-}$ MEFs in which expression of FLAG-tagged human LGP2 CTD or CTD K634E is induced following 72 h of dox treatment. β-Actin serves as loading control.

E Northern blot analysis of dsRNA-derived siRNAs in two individual clones of *Ifnar1*$^{-/-}$ iLGP2 CTD and CTD K634E MEFs left untreated or treated with dox for 24 h prior to transfection with dsRNA-GFP. Twenty-four hours post-transfection, cells were harvested and the generation of siRNAs was analysed by Northern blotting using a probe specific for dsRNA-GFP. The arrow points to dsRNA-GFP-derived siRNAs. A miRNA ladder was used as a size marker and endogenous U6 served as loading control.

Source data are available online for this figure.

© 2018 The Authors 

**Figure EV5. Expression of RIG-I or MDA5, unlike that of LGP2, does not inhibit dsRNA-mediated RNAi in *Ifnar1*$^{-/-}$ cells.**

A Doxycycline treatment does not impact on dsRNAi in parental *Ifnar1*$^{-/-}$ MEFs that lack inducible LGP2 expression constructs.

B Verification of doxycycline-dependent induction of FLAG-RIG-I (iRIG-I) and FLAG-MDA5 (iMDA5) expression in *Ifnar1*$^{-/-}$ MEFs by flow cytometry. Various *Ifnar1*$^{-/-}$ iRIG-I and iMDA5 clones were treated for 72 h with doxycycline (dox) and subsequently fixed, permeabilised and stained with a FLAG-Cy3 antibody followed by flow cytometry.

C Immunoblot analysis of four clones of *Ifnar1*$^{-/-}$ MEFs in which expression of FLAG-RIG-I or FLAG-MDA5 is induced following 72 h of doxycycline (dox) treatment. β-Actin serves as loading control.

D Expression of full-length RIG-I or MDA5 does not affect dsRNA-mediated RNAi in *Ifnar1*$^{-/-}$ cells. *Ifnar1*$^{-/-}$ iRIG-I and *Ifnar1*$^{-/-}$ iMDA5 cells, which also express a destabilised form of GFP (d2GFP), were transfected with Cy5-labelled long dsRNA corresponding to the first 200 nt of Renilla luciferase (dsRNA-RL) or GFP (dsRNA-GFP) in the absence or presence or doxycycline. Forty-eight hours post-transfection, cells were harvested and d2GFP expression in live, single, $Cy5^+$ cells was analysed by flow cytometry. Histogram plots of one representative clone are shown and are representative of three independent experiments. Each histogram and bar represents a sample size of 10,000 cells. Bar graphs display the percentage of GFP median fluorescence intensity of dsRNA-GFP-transfected cells relative to dsRNA-RL-transfected cells in four independent clones. The median fluorescence values were normalised to those in Renilla-transfected samples. Mean values and SD of three independent experiments are shown. Statistical analysis was performed using two-way ANOVA with Sidak's multiple comparisons test as post-test for pairwise comparisons. Significant differences with Sidak's multiple comparisons test are shown (ns, not significant).

Source data are available online for this figure.

 © 2018 The Authors

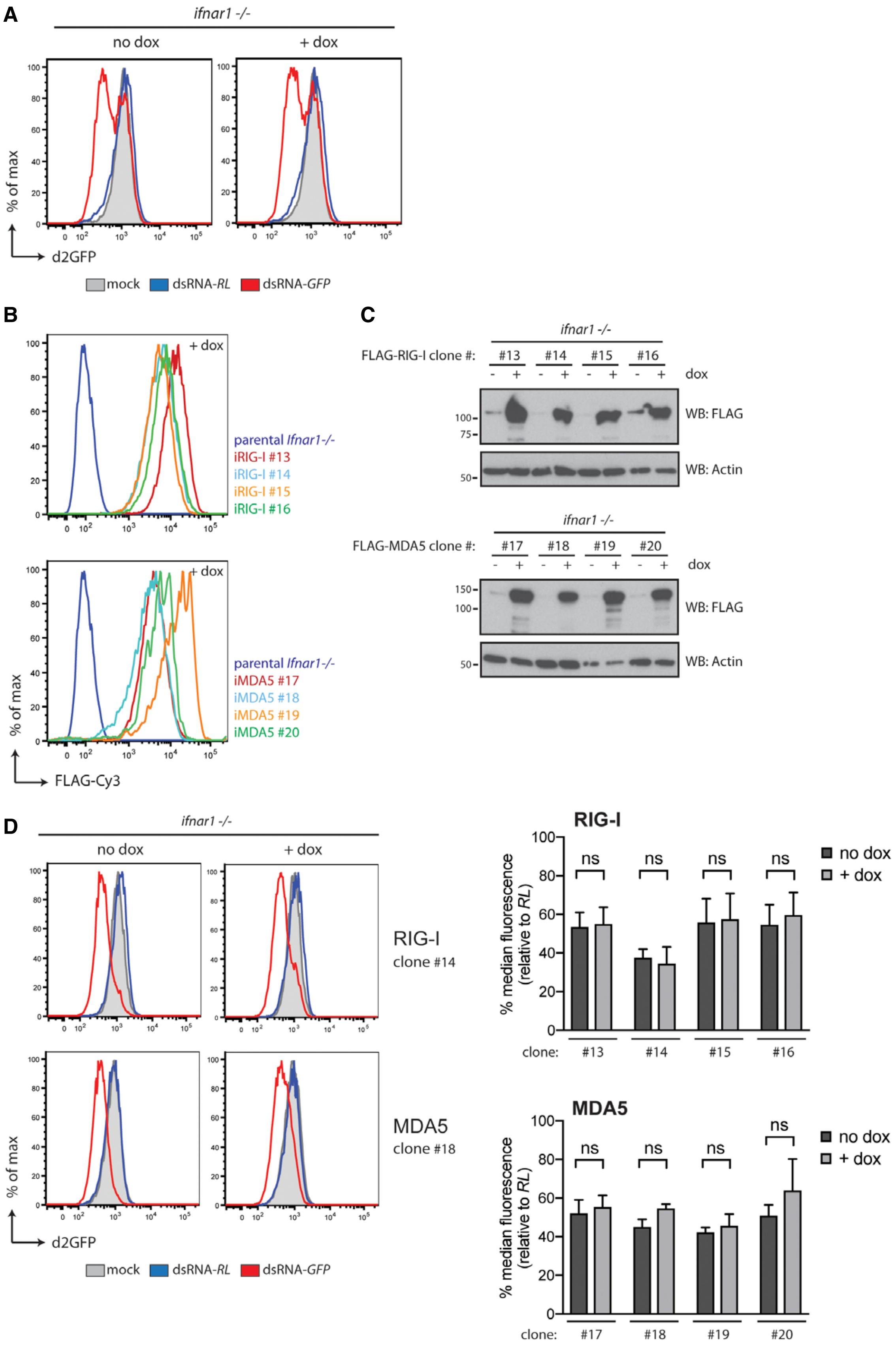
A
ifnar1 -/-
no dox
+ dox
% of max
d2GFP
mock
dsRNA-RL
dsRNA-GFP
B
+ dox
parental Ifnar1-/-
iRIG-I #13
iRIG-I #14
iRIG-I #15
iRIG-I #16
+ dox
parental Ifnar1-/-
iMDA5 #17
iMDA5 #18
iMDA5 #19
iMDA5 #20
% of max
FLAG-Cy3
C
ifnar1 -/-
FLAG-RIG-I clone #:
#13
#14
#15
#16
dox
WB: FLAG
WB: Actin
ifnar1 -/-
FLAG-MDA5 clone #:
#17
#18
#19
#20
dox
WB: FLAG
WB: Actin
D
ifnar1 -/-
no dox
+ dox
RIG-I
clone #14
MDA5
clone #18
% of max
d2GFP
mock
dsRNA-RL
dsRNA-GFP
RIG-I
% median fluorescence (relative to RL)
ns
no dox
+ dox
clone:
#13
#14
#15
#16
MDA5
% median fluorescence (relative to RL)
ns
no dox
+ dox
clone:
#17
#18
#19
#20

**Figure EV5.**

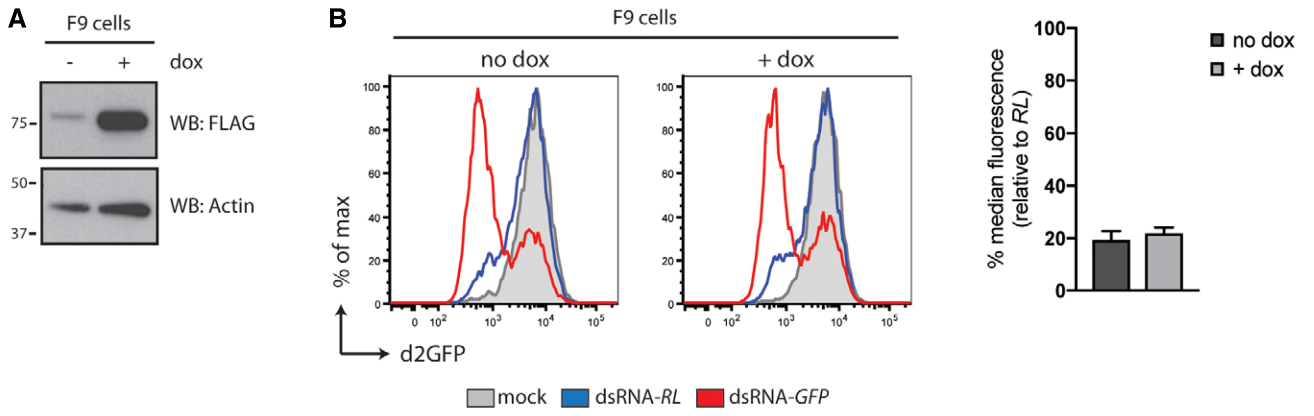


**Figure EV6. Expression of LGP2 does not affect dsRNA-mediated RNAi in F9 embryonic carcinoma cells.**

A Immunoblot analysis of F9 embryonic carcinoma cells in which expression of FLAG-tagged human LGP2 is induced following 72 h of doxycycline (dox) treatment. β-Actin serves as loading control.

B F9 iLGP2 cells were stably transduced with a lentivirus encoding a destabilised form of GFP (d2GFP) and subsequently transfected with Cy5-labelled dsRNA-RL or dsRNA-GFP in the absence or presence or doxycycline. Forty-eight hours post-transfection, cells were harvested and d2GFP expression in live, single, $Cy5^+$ cells was analysed by flow cytometry. Histogram plots of one representative experiment are shown and represent a sample size of 10,000 cells. Bar graphs display the percentage of GFP median fluorescence intensity of dsRNA-GFP-transfected cells relative to dsRNA-RL-transfected cells. Mean values and SD of two independent experiments with duplicate samples are shown.

Source data are available online for this figure.

 © 2018 The Authors
