# Supplementary material for: The RIG‐I‐like receptor LGP2 inhibits Dicer‐dependent processing of long double‐stranded RNA and blocks RNA interference in mammalian cells
Source: EMBO J. 2018 Jan 19;37(4):e97479. doi: 10.15252/embj.201797479 (PMC5813259; doi:10.15252/embj.201797479)
Supplement: Supplementary file 2 — Source Data for Expanded View [file EMBJ-37-e97479-s006.zip › 97479_Source_Data_for_EV_Figures/Source_Data_for_FigEV4.pdf]

D.

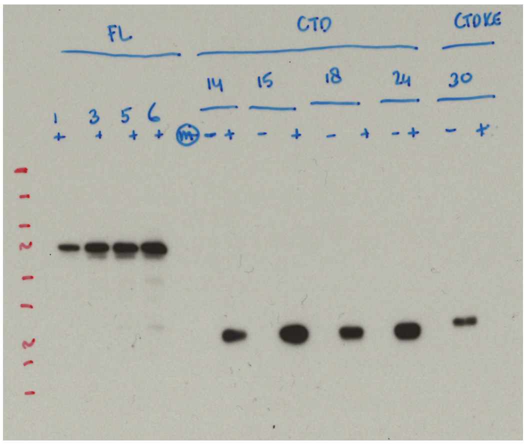

WB FLAG

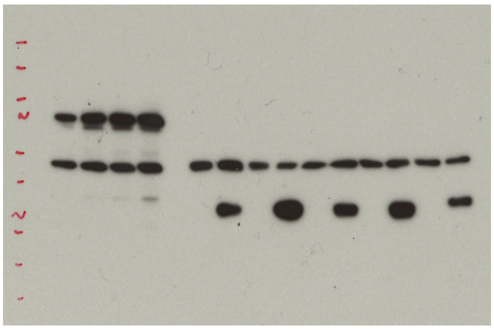

WB Actin (after FLAG)

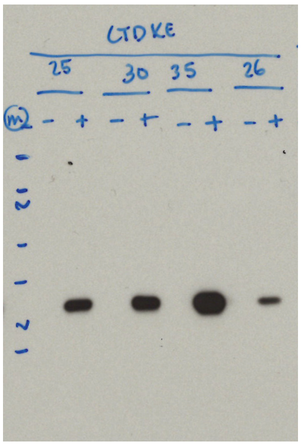

WB FLAG

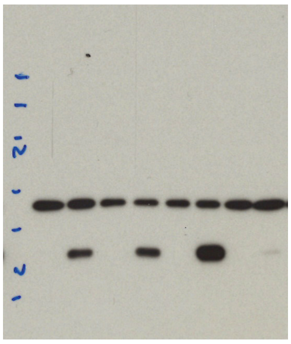

WB Actin (after FLAG)

E.

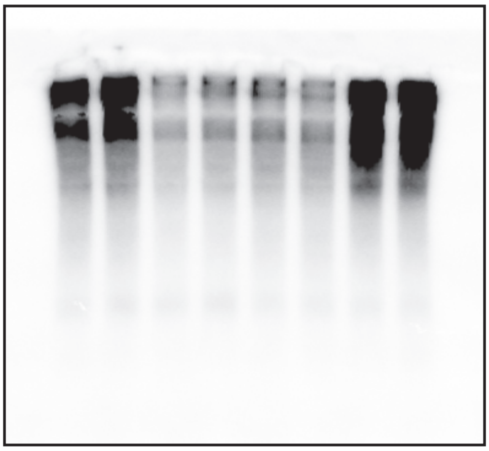

GFP probe

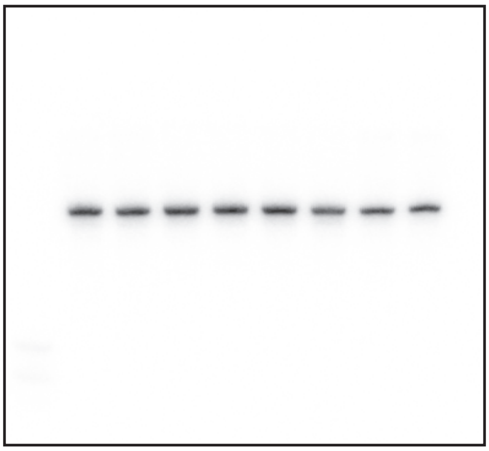

miRNA marker + U6 probe (used for U6)

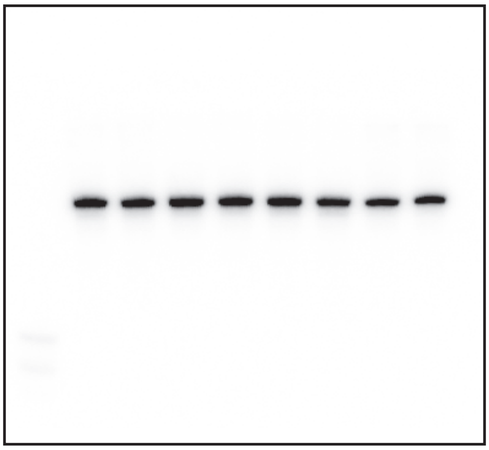

miRNA marker + U6 probe
(used for miRNA marker, far left)
