# Supplementary figures and images for: The RIG‐I‐like receptor LGP2 inhibits Dicer‐dependent processing of long double‐stranded RNA and blocks RNA interference in mammalian cells
Source: EMBO J. 2018 Jan 19;37(4):e97479. doi: 10.15252/embj.201797479 (PMC5813259; doi:10.15252/embj.201797479)

A.

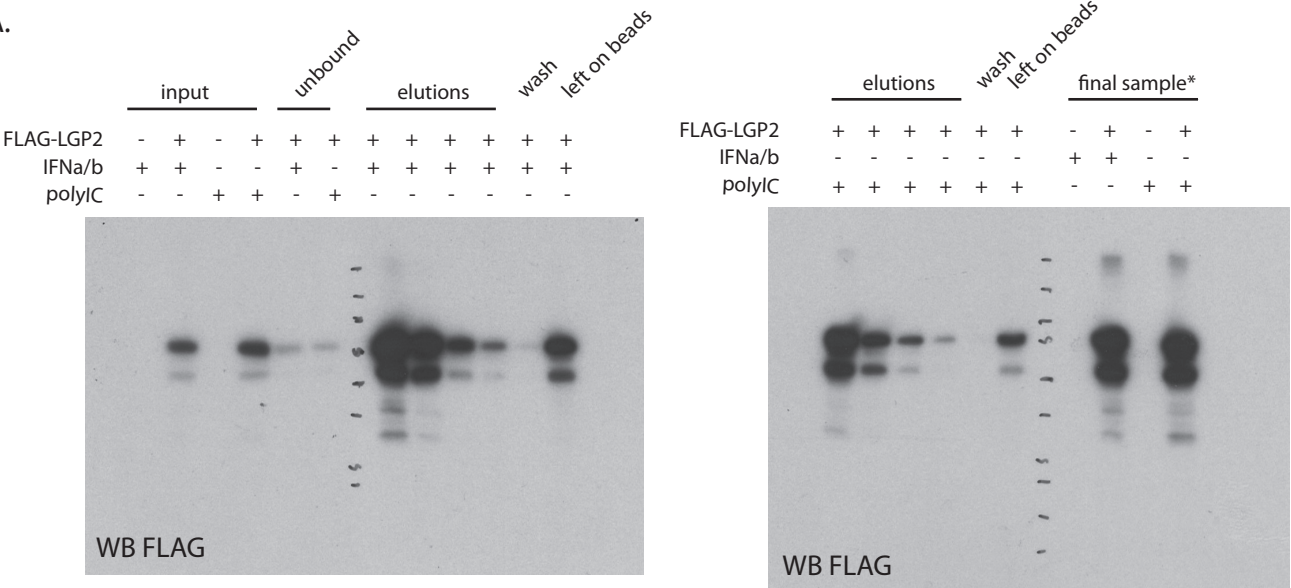

B.

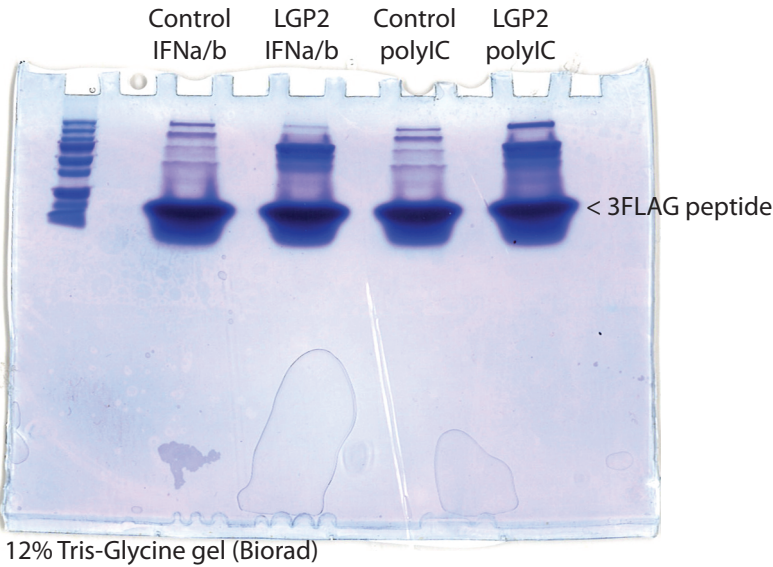

Supplement: Supplementary file 2 — Source Data for Expanded View [file EMBJ-37-e97479-s006.zip › 97479_Source_Data_for_EV_Figures/Source_Data_for_FigEV1.pdf]

A.

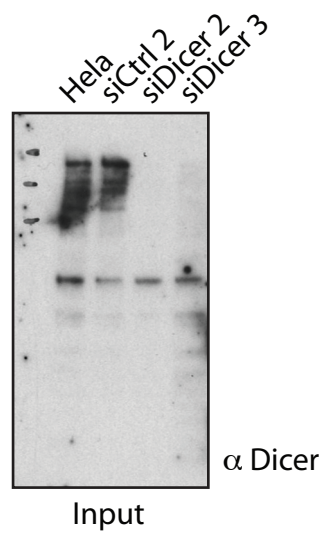

B.

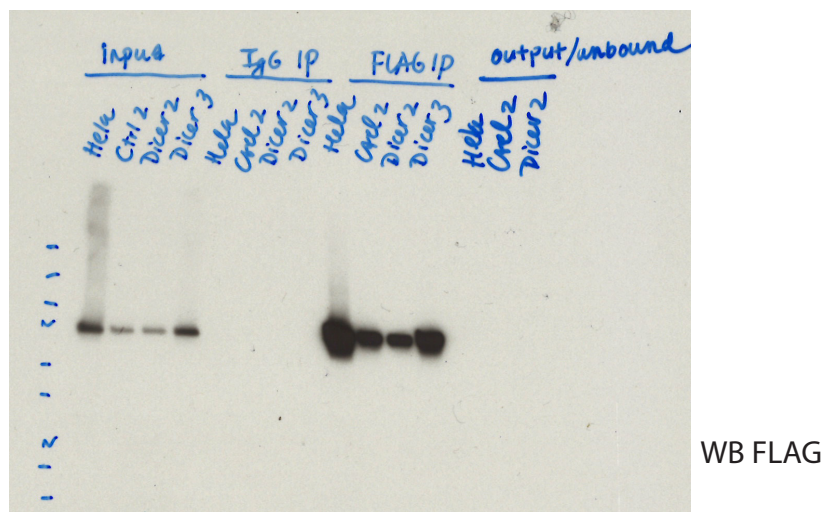

Supplement: Supplementary file 2 — Source Data for Expanded View [file EMBJ-37-e97479-s006.zip › 97479_Source_Data_for_EV_Figures/Source_Data_for_FigEV2.pdf]

C.

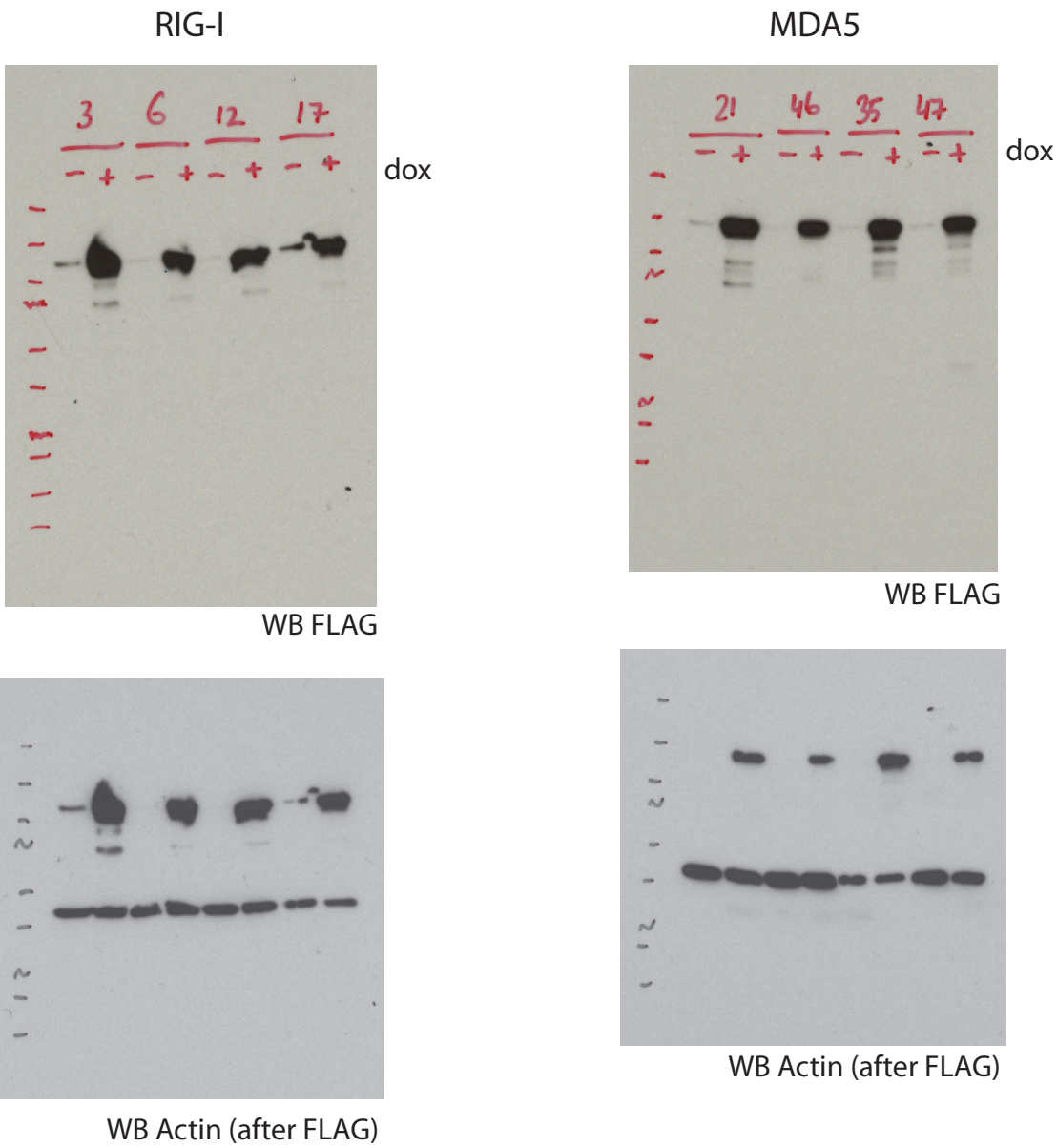

Supplement: Supplementary file 2 — Source Data for Expanded View [file EMBJ-37-e97479-s006.zip › 97479_Source_Data_for_EV_Figures/Source_Data_for_FigEV5.pdf]

A.

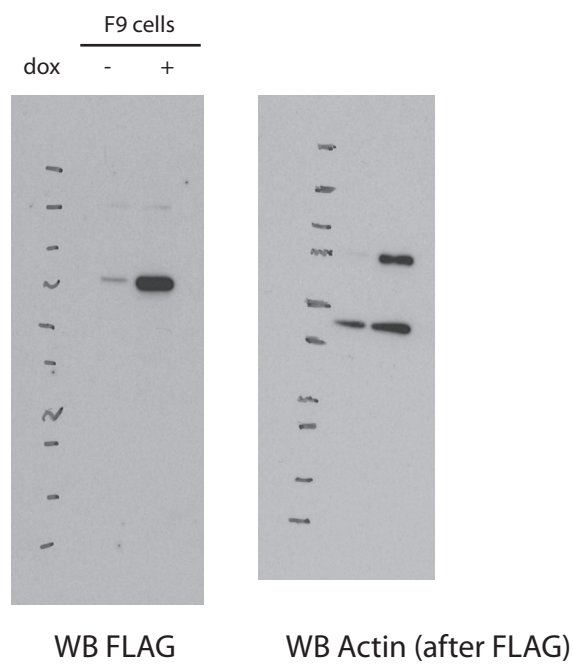

Supplement: Supplementary file 2 — Source Data for Expanded View [file EMBJ-37-e97479-s006.zip › 97479_Source_Data_for_EV_Figures/Source_Data_for_FigEV6.pdf]

C.

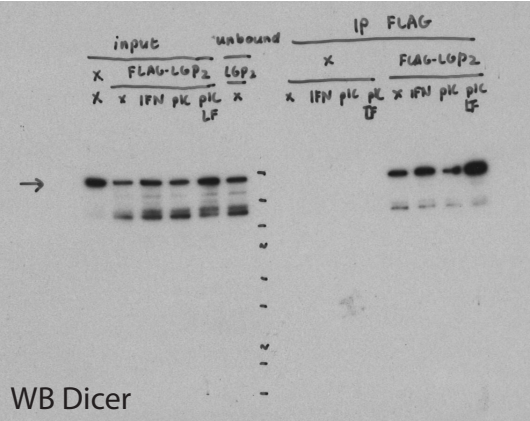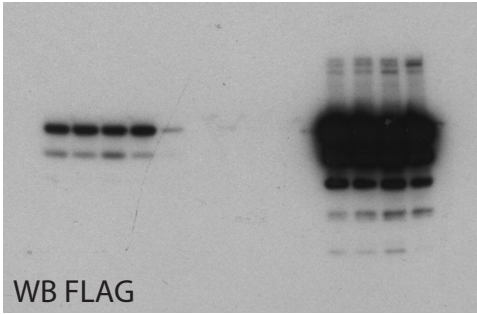

D.

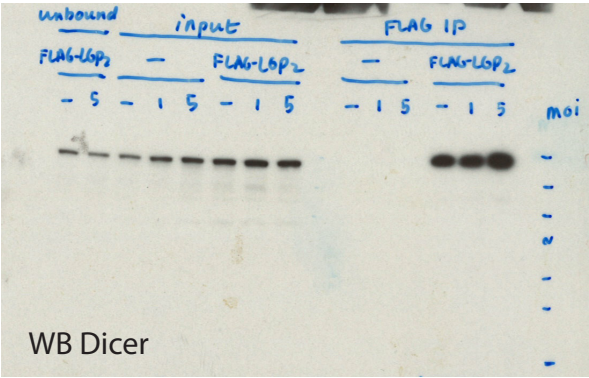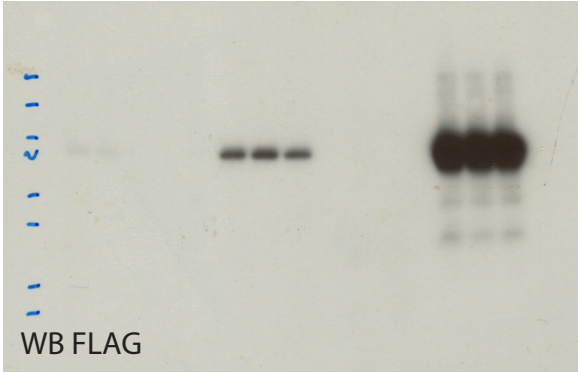

E.

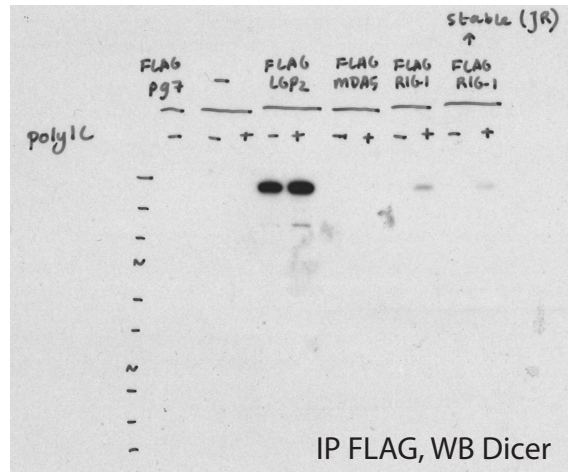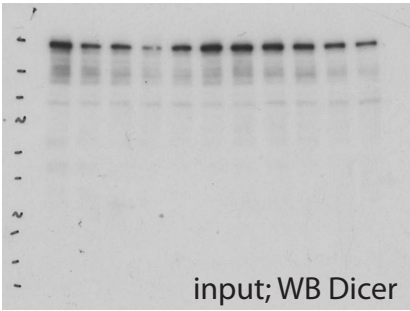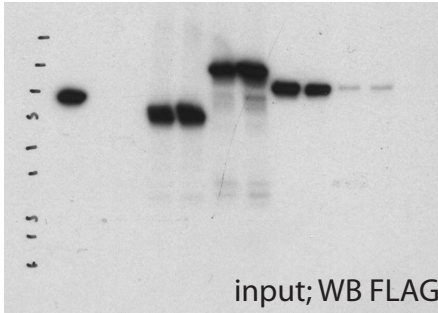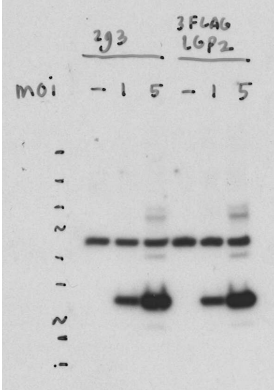

G.

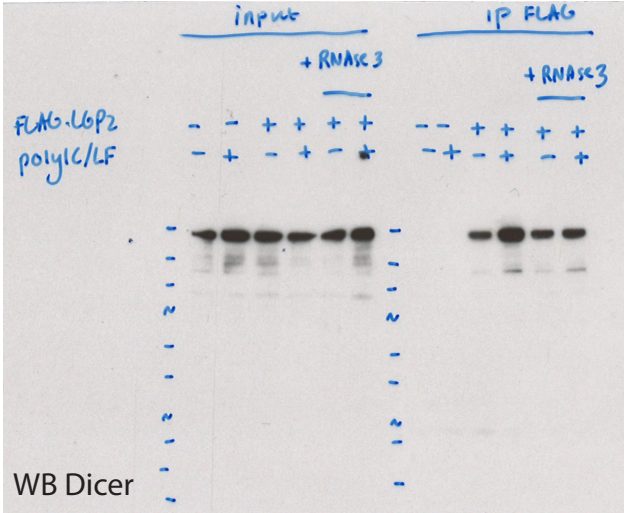

IP samples

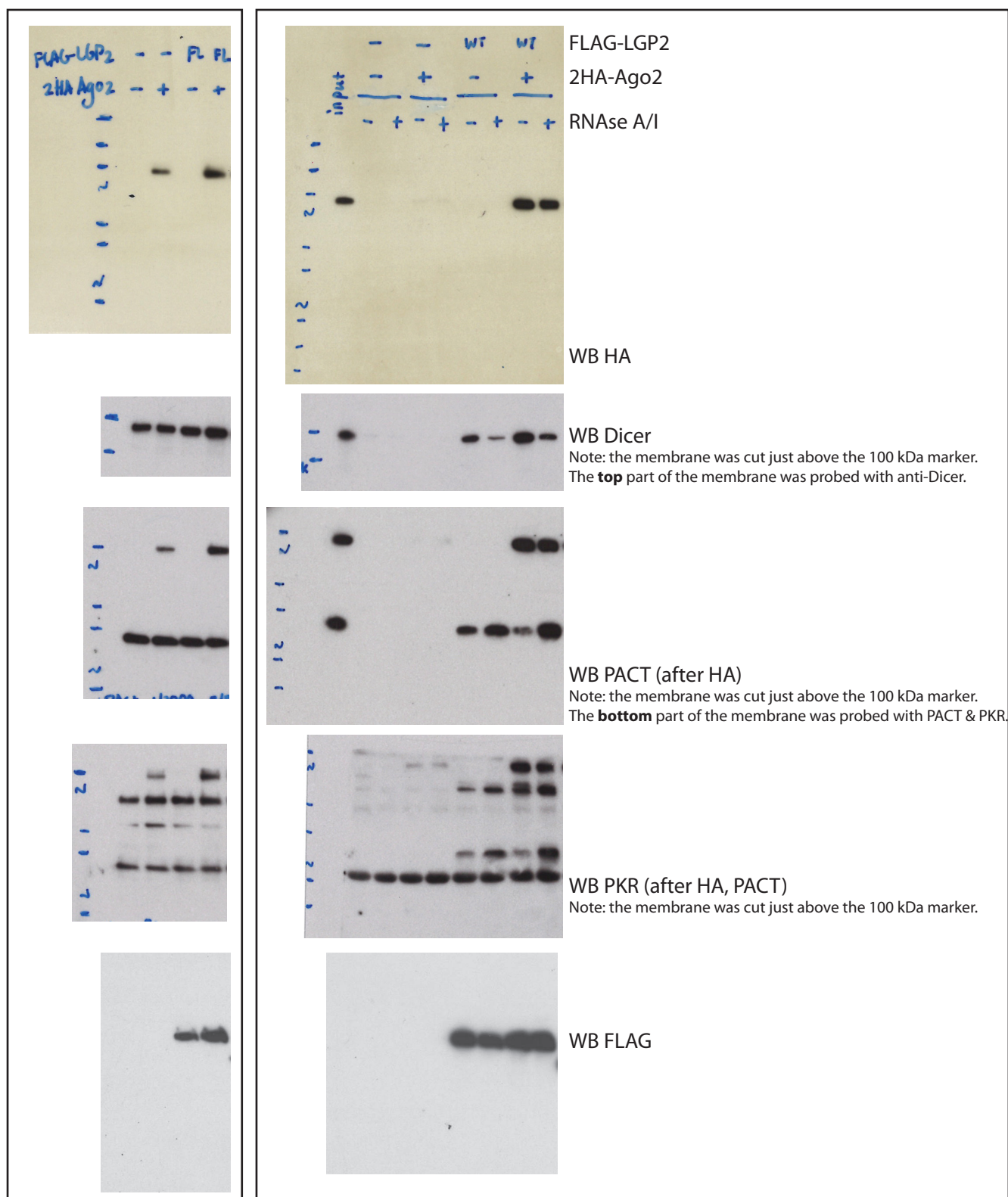

Supplement: Supplementary file 4 — Source Data for Figure 1 [file EMBJ-37-e97479-s002.pdf]

B.

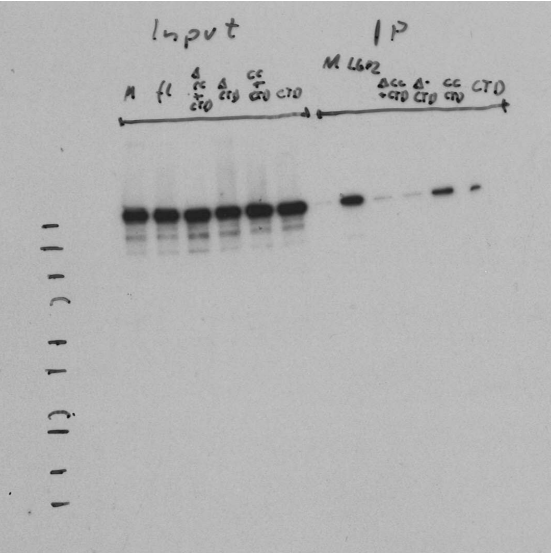

C.

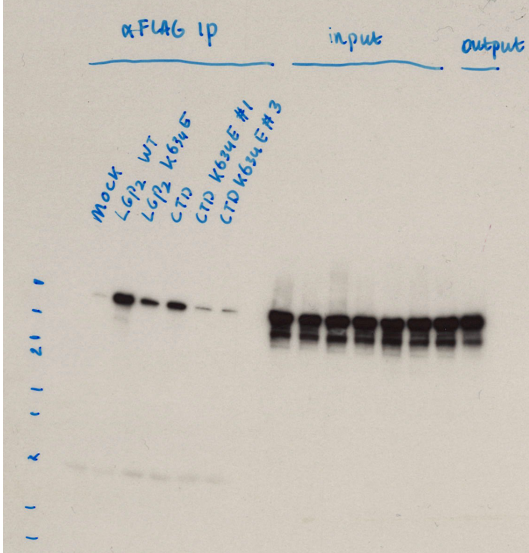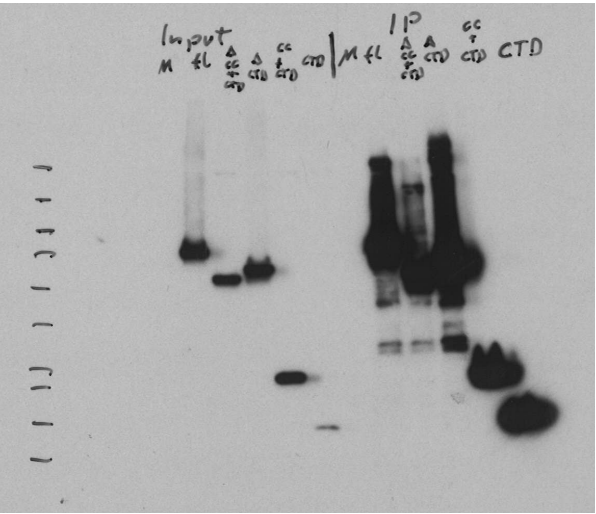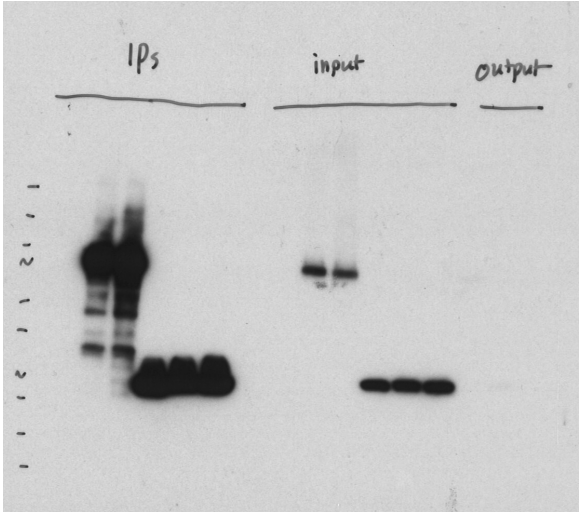

Supplement: Supplementary file 5 — Source Data for Figure 2 [file EMBJ-37-e97479-s003.pdf]

A.

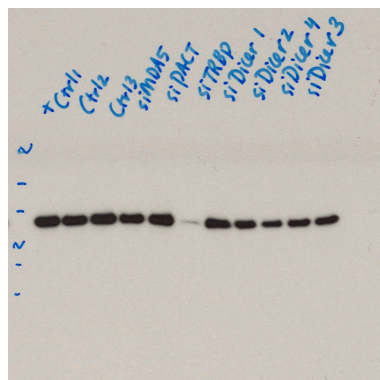

WB PACT

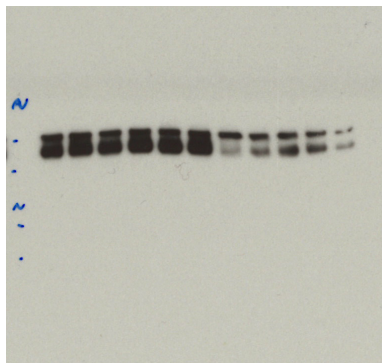

WB TRBP

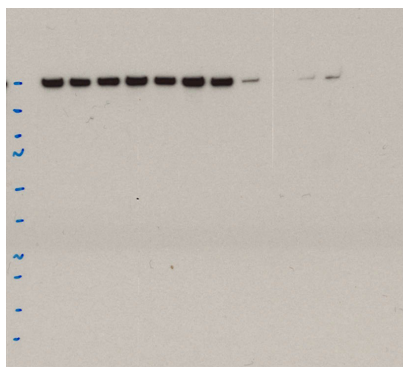

WB Dicer

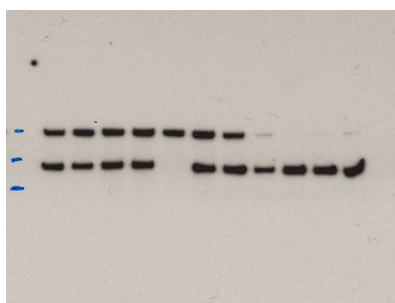

WB MDA5 (probed after WB Dicer)

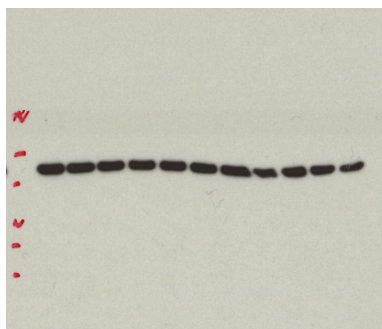

WB Actin

Supplement: Supplementary file 6 — Source Data for Figure 3 [file EMBJ-37-e97479-s004.pdf]

A.

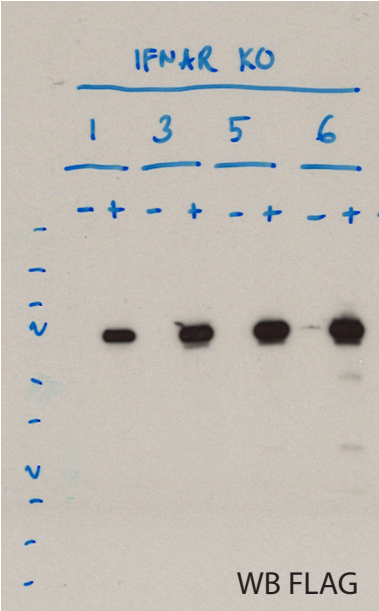

Note: clones were renamed clone 1, 2, 3, 4 when preparing the manuscript

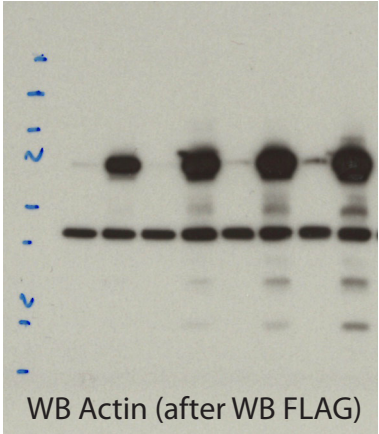

B.

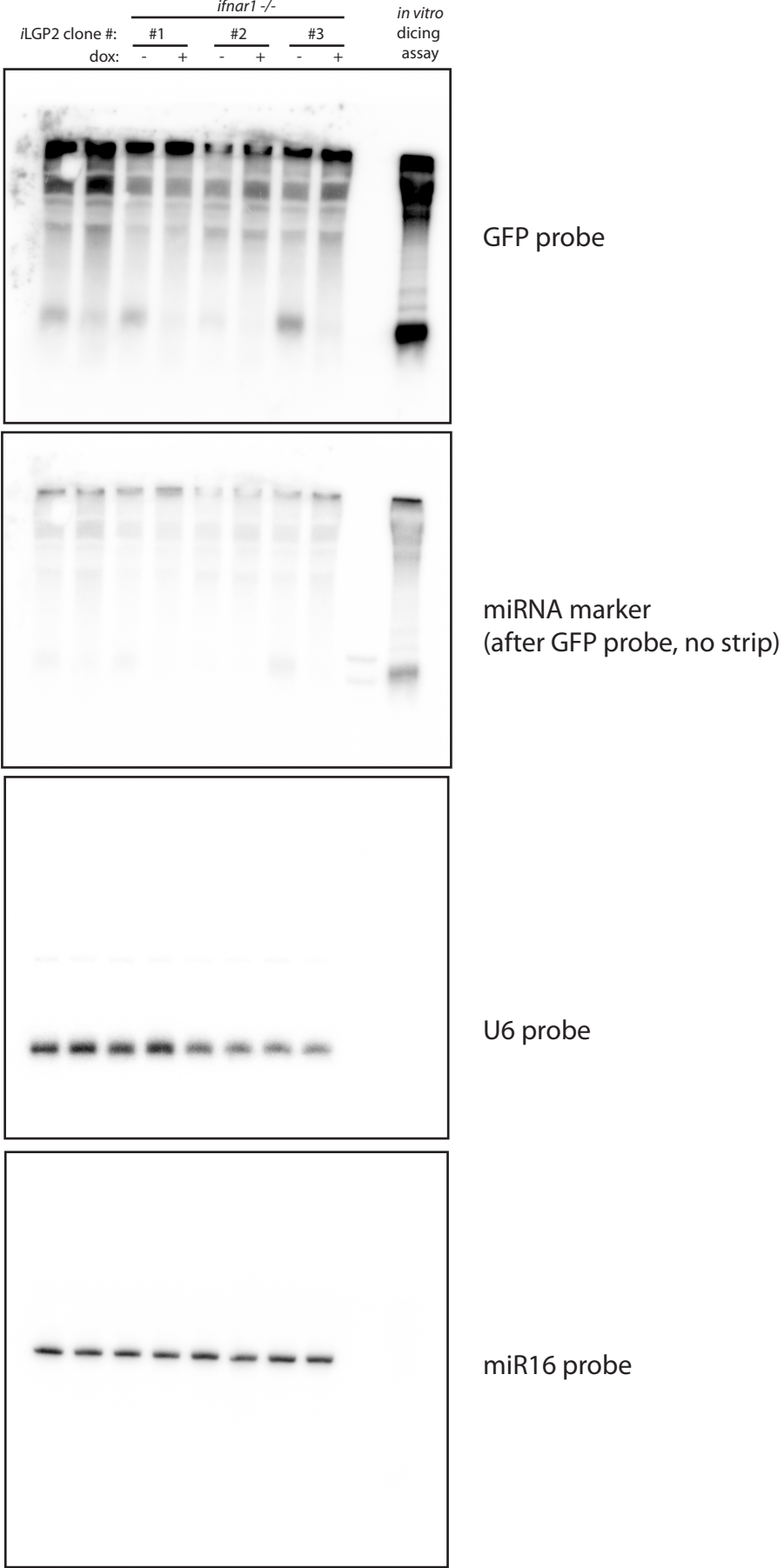

Supplement: Supplementary file 7 — Source Data for Figure 5 [file EMBJ-37-e97479-s005.pdf]
